# Skins Comparative Analysis of Collagen Functionality and Peptide Bioactivities from Yak, Cattle, and Donkey Skins

**DOI:** 10.3390/foods14213776

**Published:** 2025-11-04

**Authors:** Yaoyuan Kuai, Yufeng Duan, Xue Yang, Ruheng Shen, Wen Wang, Li Zhang, Long He, Cheng Chen, Xiaojin Yuan, Xiangmin Yan, Hongbo Li

**Affiliations:** 1 College of Food Science and Engineering, Gansu Agricultural University, Lanzhou 730070, China; 2 Institute of Animal Husbandry, Xinjiang Academy of Animal Husbandry, Urumqi 830011, China

**Keywords:** variety, collagen, emulsification, functional properties

## Abstract

Collagen peptides derived from animal skins are valuable bioactive ingredients with diverse nutritional and functional properties. This study systematically compared the nutritional value, collagen structure function properties, and bioactivities of collagen peptides from six types of animal skins, including yak skins from different altitudes, Pingliang Red cattle skin, Xinjiang Brown cattle skin, and donkey skin. In terms of nutritional value, low-altitude yak skin contained 34.15 g/100 g protein and 1.78 g/100 g fat, exhibiting superior overall performance compared with other samples. Regarding structure–function relationships, low-altitude yak skin showed the highest emulsifying activity (12.05 m^2^/g) and foaming capacity (26%), which were attributed to its smaller particle size and higher surface hydrophobicity, whereas mid-altitude yak skin demonstrated greater thermal stability (115.3 °C) and a more compact microstructure. In terms of bioactivity, yak leather contains 23,558 to 25,966 peptides, with relatively high activity of antibacterial peptides and anti-diabetic peptides. Pingliang red cowhide and Xinjiang brown cowhide contain 1515 and 2186 polypeptides, respectively, which have strong antihypertensive activity. The antibacterial effect of donkey skin is more obvious, with a total peptide count of 11,678. Collectively, these findings reveal significant differences in the nutritional and processing-related properties of the six skin types and provide potential evidence for expanding their applications in the field of functional foods.

## 1. Introduction

Collagen, as an important natural functional protein, its source differences affect its physical and chemical properties and its application behavior in food systems. Currently, collagen used in the food industry mainly comes from animal by-products such as pig skin, cowhide, fish skin, and chicken cartilage [1]. Collagen proteins from different sources exhibit fundamental differences in amino acid composition, triple helix structural stability, and molecular cross-linking density, leading to significant variations in functional properties such as solubility, thermal stability, emulsification capacity, and gelling ability. For example, fish-derived collagen proteins have lower levels of proline and hydroxyproline, poor thermal stability, and are suitable for low-temperature processed foods such as functional beverages and frozen desserts [2]. Mammalian collagen, on the other hand, is suitable for meat products and candies that require heating processing, and can effectively improve texture and water retention capacity [3]. Therefore, understanding the intrinsic differences among collagen sources is essential for tailoring their functional properties to specific food applications.

Cowhide is one of the most widely used animal-derived raw materials for industrial collagen production. In regions rich in agricultural and livestock resources, its abundant availability, convenient collection, and dense tissue structure make it a critical and sustainable source for collagen extraction. Notably, cowhide is rich in type I collagen, which contributes to its high thermal stability, strong gelling capacity, and excellent film-forming and emulsifying properties [4]. These physicochemical characteristics endow bovine hide collagen with remarkable functional advantages, particularly in food processing applications. For instance, it plays a significant role in improving the texture, water retention, and structural integrity of dairy products and meat-based items [5]. Moreover, its compatibility with bioactive ingredients and ability to form stable colloidal systems make it especially suitable for use in functional beverages and health-oriented food formulations. Therefore, bovine hide not only holds great promise for value-added utilization but also aligns with current trends in sustainable food ingredient development and functional food innovation.

In addition, it is noteworthy that donkey skin is rich in nutrients such as collagen and amino acids. Its relatively high collagen content, together with favorable structural and physicochemical properties, makes it more suitable for the preparation of tonic products, although extensive studies have already focused on the extraction and application of collagen from cattle skin. For example, Gao et al. [6] employed high-pressure processing to extract gelatin from cowhide as a fat replacer in food, emphasizing its potential to improve texture and reduce fat content without compromising sensory quality. Meanwhile, Ma et al. [7] acid-treated bovine hide to induce sufficient swelling and subsequently extracted collagen fibers to fabricate collagen-based films, which exhibited favorable mechanical strength and biodegradability. Although previous studies have investigated the physicochemical properties of collagens from various sources [8], the structural characteristics and functional differences in yak skin collagen remain insufficiently understood, and the relationship between its structure and function requires further investigation. In this study, yak, cattle, and donkey skins were selected as representative raw materials to investigate the influence of species and environmental factors on collagen characteristics. Yak, native to high-altitude regions such as the Qinghai–Tibet Plateau, possesses unique physiological adaptations that may affect its collagen composition and functionality. In contrast, cattle and donkey skins, commonly obtained from low-altitude regions, exhibit distinct structural and technological properties. Such research is crucial for elucidating the differences in processing and performance among these collagen sources and for facilitating the development of distinctive functional and high-value products from animal by-products.

Therefore, we hypothesize that differences in species and environmental factors (such as altitude) lead to distinct nutritional composition, collagen structural characteristics, and peptide bioactivities among yak, cattle, and donkey skins. To verify this hypothesis, this study systematically compared the nutritional value, collagen structure–function properties, and collagen peptide bioactivities of six types of animal skins (including yak skins from different altitudes, Pingliang Red cattle skin, Xinjiang Brown cattle skin, and donkey skin). These findings provide a theoretical basis for optimizing the utilization of bovine skin resources and offer scientific support for the development of high value-added collagen and peptide products.

## 2. Materials and Methods

### 2.1. Materials and Chemicals

All animal experiment procedures have been reviewed and approved by the Animal Ethics and Use Committee of Gansu Agricultural University (Approval Number: GSAU-Eth-FSE-2024-003).

The skins of yaks at low, medium and high altitudes are, respectively, sourced from local large-scale slaughterhouses (Xining, Haibei and Yushu, China) and are called LA-YS, MA-YS and HA-YS. Pingliang Red cattle skins are sourced from large local slaughterhouses (Pingliang, China) and are referred to as PLRS. Xinjiang brown cattle skins are sourced from large slaughterhouses (Yili, China) and are referred to as XJBS. Donkey skins are sourced from large slaughterhouses (Qingyang, China) and are referred to as DS. Phosphate-buffer solution was from Lanjieke Technology Co., Ltd. (Hefei, China), and pepsin (3000 U/mg) was from Maclean’s Biochemical Technology Co., Ltd. (Shanghai, China).

### 2.2. Pretreatment of Cowhides

**Figure 1:**
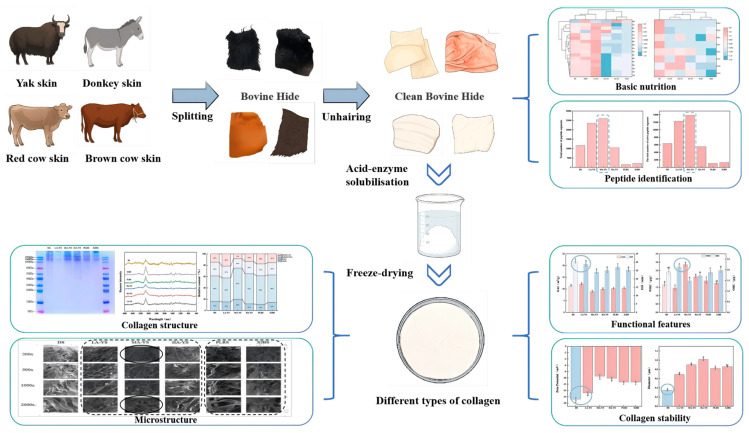
Experimental design process chart.

Split the cowhide into uniform blocks and pack them in self-sealing bags and store them at −18 °C for spare. Before each use, put it in 0–4 °C to thaw for 10–12 h, after thawing, scrape off the cow’s hair and the fat on the back to be used. The entire experimental design process is shown in Figure 1.

### 2.3. Determination of Conventional Nutrients

Determination of moisture, protein, fat and ash content were determined by referring to the methods in GB 5009.3-2016 Determination of Moisture in Foods [9], GB 5009.5-2016 Determination of Protein in Foods [10], GB 5009.6-2016 Determination of the Total Fat Content of Meat and Meat Products [11], GB 5009.4-2016 Determination of Ash in Foods [12], respectively. And the hair in the sample was removed, and the sample was divided into small pieces to ensure that it could be fully reacted, and the consistency of the sample was maintained.

### 2.4. Determination of Amino Acids

The amino acid profile of the cowhides was determined according to the methodology by [13]. Weigh 1 g of sample, add 10 mL 1:1 hydrochloric acid solution in the hydrolysis tube, mix well, put the hydrolysis tube in the 110 ± 1 °C electric blast thermostat in the hydrolysis of 22 h, take out, cool to room temperature. Open the hydrolysis tube, filter the hydrolysis solution into a 25 mL volumetric flask, rinse the hydrolysis tube with a small amount of water for several times, the water wash solution was transferred to the same 25 mL volumetric flask, and finally, the water was fixed to the scale, shaking well. Accurately sucked 0.5 mL filtrate into a 15 mL test tube, evaporated with a constant temperature water bath, and then fixed to 10 mL with 0.02 mol/L hydrochloric acid solution, shaken and mixed well, then passed through 0.22 μm microporous filter membrane, and then analyzed by amino acid analyzer (LA8080, Hitachi High-Technologies Co., Ltd., Tokyo, Japan).

### 2.5. Determination of Fatty Acids

Determination of amino acids in cowhides according to the method described by Sims et al. [14]. Weigh 5 g of the sample into a 100 mL colorimetric tube, add 8 mL of water, mix well, add 10 mL of hydrochloric acid, mix well. The flask was hydrolyzed in a water bath at 80 °C for 1 h. After hydrolysis of the sample, 10 mL of 95% ethanol was added and mixed well. The fat was extracted three times with 100 mL of ether, and the combined extracts were transferred into a 100 mL flat-bottomed flask, where the ether layer was evaporated to yield the fat. In the fat extract, continue to add 4 mL 2% sodium hydroxide methanol solution, water bath in 45 °C water bath for 30 min, add 4 mL 14% boron trifluoride methanol solution, water bath in 45 °C water bath for 30 min. after the completion of the water bath, wait for the temperature to drop to room temperature, in the centrifugal tube add 3 mL n-hexane, after shaking the extraction for 2 min, let stand and wait for the stratification. The supernatant was filtered through 0.45 μm membrane and analyzed by gas chromatography analyzer (7890A, Agilent Technologies, Inc., Santa Clara, CA, USA)

### 2.6. Extraction of Collagen

The extraction of collagen from cowhide was referred to the acid method of He et al. [15] with slight modification. The cowhides were cut into 1 cm^2^ pieces and soaked in 5% sodium carbonate solution (1:15) for 18 h to degrease; the cowhide samples were soaked in 5% sodium chloride solution at 4 °C for 12 h to remove non-collagenous proteins; the cowhides were washed repeatedly with deionized water to obtain clean cowhides; the clean cowhides were soaked in 0.5 M acetic acid at 4 °C for 24 h at a ratio of 1:10 (*w*/*v*) and then homogenized using a high-speed tissue grinder at 12,000 rcf The homogenized solution was mixed with 0.5 M acetic acid (20:1) and pepsin (E/S, 4%) on a magnetic stirrer for 12 h, then centrifuged at 10,000× *g* rcf for 10 min to recover the supernatant. Collagen was collected after salting out (final NaCl concentration of 0.9 mol/L) and dissolved in acetic acid (0.5 M), and the collagen samples were dialyzed in distilled water at 4 °C for 48 h. The molecular cutoff of the dialysis bag was 3500 Da (Jielepu, Hunan, China), and the dialyzed samples were lyophilized and stored at −18 °C for subsequent analysis.

### 2.7. Thermal Stability of Collagen

Referring to the method of Wei et al. [16], 8 mg of collagen was placed in a solid crucible and heated from 20 to 250 °C under nitrogen atmosphere (purity ≥ 99.9%) at a specified heating rate of 10 °C/min to plot absorption and exothermic curves as well as thermogravimetric measurements (DSC600, Hitachi High-Technologies Co., Ltd., Tokyo, Japan). The blank control was an empty crucible, and the determination of both collagens was repeated three times under the same conditions.

### 2.8. Collagen Molecular Weight Determination

The molecular weight distribution of collagen was studied using sodium dodecyl sulfate-polyacrylamide gel electrophoresis (SDS-PAGE). Following the method of Matinong et al. [17] was slightly modified to use 5% concentrated gel and 10% separated gel for electrophoresis. The sample mass concentration of 3 mg/mL collagen solution was configured with 0.5 mol/L acetic acid and the up-sampling buffer (0.01 g SDS, 2.5 mL glycerol, 0.07268 g Tris base, 0.5 mL 2-mercaptoethanol, 0.01g bromophenol blue, pH adjusted to 6.8, and fixed to 10 mL) was blended at a 4:1 ratio and subjected to boiling water for 3 min. The sample was heated in boiling water for 3 min (turning dark blue), cooled and centrifuged at 12,000× *g* rcf for 2 min, and the supernatant 20 μL of protein sample was added into the lane. Electrophoresis was run at 80 V for the stacking gel, while 100 V was applied for the separating gel. At the end of electrophoresis, the film was stained with Cauloblue R-250 and decolorized with ethanol–acetic acid decolorizing solution and finally observed and analyzed with a gel imager.

### 2.9. Raman Spectra

The method was slightly modified based on that of Liang et al. [18]. The secondary structures of the extracted collagen were measured and analyzed using a Raman spectrometer (LabRAM HR-800, HORIBA Jobin Yvon, Paris, France), with the spectral scanning range set to 400–4000 nm^−1^. After the determination, the spectral bands of the amide 1 were analyzed using the PeakFit 4.12 software (Sea Solve Software, Inc., San Jose, CA, USA) was conducted to evaluate the relative percentage of various secondary structures present in the collagen samples.

### 2.10. Ultraviolet-Visible Spectrum (UV)

The ultraviolet-visible absorption spectra of collagen were determined according to the method of Liu et al. [19]. The collagen samples obtained from extraction were placed in 0.5 mol/L acetic acid solution, dissolved to 0.5 mg/mL solution, diluted 10-fold and then scanned in the near-ultraviolet region of 200–400 nm using a UV-visible spectrophotometer (UV-2600, Shimadzu Manufacturing, Kyoto, Japan) to obtain the wavelength of the UV maximal absorption of the collagen solution.

### 2.11. Scanning Electron Microscope (SEM)

Observe the microstructure of different collagens by referring to the method of Duan et al. [20]. The lyophilized cowhides collagen samples were placed on the sample stage glued with conductive tape with tweezers, respectively, to avoid the overlapping of protein particles affecting the structural observation, vacuum sprayed with gold, and placed on the scanning electron microscope to observe the surface morphology and structure of the collagen samples (EM6900, Beijing Zhongke Keji Co., Ltd., Beijing, China).

### 2.12. Emulsification Activity and Emulsion Stability

The method described by Yu et al. [21] was adopted with minor modifications. To determine the emulsification activity index (EAI) and emulsion stability index (ESI) of collagen, 3 mL of peanut oil and 9 mL of collagen solution (30 mg/mL) were added into a 50 mL centrifuge tube, and emulsification was carried out by shear emulsification using a high-speed shear instrument (HR-250D, Shanghai HUYI Science and Technology Co., Ltd., Shanghai, China) at a speed of 10,000 r/min for 1 min. At the end of emulsification, the emulsion was diluted 100-fold with 0.1% SDS at 0 and 10 min. The diluted mixture was mixed thoroughly for 10 s by a vortex mixer (XW-80A, Jiangsu Qilinbeier Instrument Manufacturing Co., Ltd., Nantong, China). The absorbance values were measured at 500 nm with 0.1% SDS as blank control and recorded as A0 and A10, respectively.

(1) $$\mathrm{EAI}\;(m^{2}/g)=\frac{\left(2\;\times \;2.303\;\times \;A\;\times \;\mathrm{DF}\right)}{\left(I\;\times \;Ø\;\times \;C\;\times \;10,000\right)}$$

where A is the absorbance value of the emulsion at 500 nm for 0 min, DF is the dilution factor, I is the path length of the cuvette, Ø is the volume fraction of the oil phase (*v*/*v*), and C is the protein concentration in the aqueous phase mg/mL.

(2) $$\mathrm{ESI}\;(\min)={\;A}_{0}/(\Delta A\;\times \;\Delta t)$$

where A_0_ and A_10_ are the absorbance values of the emulsion at 500 nm for 0 min and 10 min, respectively, ΔA = A_0_ − A_10_, Δt = 10 min.

### 2.13. Emulsion Microscope

The microstructure of collagen emulsion was observed with a slight modification based on the method of Feng et al. [22]. Refer to Section 2.12 for the preparation of emulsion, take 20 μL of the prepared emulsion, place it on a clean slide, mix it well, cover the cover slip, and observe it under microscopes of different magnifications.

### 2.14. Water-Holding and Oil-Holding Properties

Water-holding and oil-holding properties were slightly modified with reference to the method of Li et al. [23].

Water-holding property: 1 g of collagen was placed in a centrifuge tube with 20 mL of pre-cooled ultrapure water, mixed in a vortex mixer, and left to stand for 20 min. The mixture was then centrifuged at 6000× *g* rcf for 10 min at 4 °C, the supernatant was removed, and the total weight of the centrifuge tube and the precipitate after centrifugation was weighed.

(3) $$\mathrm{WHC}\;(g/g)=(W_{2}-{\;W}_{1})/W_{0}$$

where W_0_ collagen dry weight (g), *W*_1_ total weight of the tube and collagen (g) and W_2_ is the total weight of the sediment and tube after centrifugation (g).

Oil-holding property: 1 g of collagen was mixed with 10 mL of peanut oil, left to stand for 20 min, and centrifuged at 6000× *g* rcf for 10 min at 4 °C, the supernatant was removed, and the total weight of the centrifuge tube and the precipitate after centrifugation was weighed.

(4) $$\mathrm{OAC}\;(g/g)=(W_{2}-W_{1})/W_{0}$$

where W_0_ collagen dry weight (g), W_1_ total weight of the tube and collagen (g) and *W_2_* is the total weight of the precipitate and the tube after centrifugation (g).

### 2.15. Determination of Particle Size and Potential 

After diluting 10.0 uL of freshly prepared collagen pure water to 1.0 mL [24], the particle size and zeta potential were determined using a Malvern particle size analyzer (Mastersizer 3000, Malvern Instruments Ltd., Worcestershire, UK).

### 2.16. Determination of Turbidity

The absorbance value of the collagen solution was measured every 10 min at 310 nm by ultraviolet spectrophotometry [25]. The absorbance value reflects the change in turbidity. The operation was repeated until the data stabilized.

### 2.17. Determination of Surface Hydrophobicity

Bromophenol blue (BPB) was used to determine hydrophobicity, following the method of Xu et al. [26] 80 µg of BPB was added to 2 mL of MPs solution at a concentration of 1 mg/mL and mixed thoroughly. The mixture was then centrifuged at 6000× *g* for 10 min. the supernatant was collected, diluted 10-fold, and the absorbance was measured at 595 nm (A_1_). Phosphate-buffer solution was used as a blank (A_2_).

(5) $$\mathrm{BPB}\;\mathrm{binding}\;(µg)\;=80\;\times \;(A_{1}-\;A_{2})/A_{2}$$

### 2.18. Foaming Property

Referring to the method of Zheng et al. [27] 5 g of cowhides collagen was accurately weighed and dissolved in deionized water (100 mL), and adjust the PH to 7.4. Transfer 20 mL of the solution was transferred to a 50 mL graduated flat-bottomed glass tube, and then the solution was stirred by a high-speed shear (HR-250D, Shanghai HUYI Co., Ltd., Shanghai, China) at a rotational speed of 10,000 r/min for one minute, and then the liquid level height (V_0_) was recorded, and the height of the liquid level was recorded again after 30 min. The height of the liquid level was recorded at this time (V_0_), and the height of the liquid level was recorded again after 30 min (V_30_), FA is the foaming ability and FS is the foaming stability. The formula is as follows:

(6) $$\mathrm{FA}\;(\%)\;=\;\frac{V_{0}\;-\;20}{20}\;\times \;100$$

(7) $$\mathrm{FS}\;(\%)=\frac{V_{30}-\;20}{V_{0}-20}\;\times \;100$$

### 2.19. Prediction of Collagen Activity

The samples were dissolved and ultrafiltered using a 10 Kd ultrafiltration tube (Merck KGaA, Darmstadt, Germany); desalted using a Waters SEP-PAK C18 solid phase extraction column, lyophilized (Waters Corporation, Milford, CT, USA); re-dissolved in formic acid and water, centrifuged, and the supernatant was taken for detection.

Liquid A used in the liquid phase was 0.1% formic acid aqueous solution, and liquid B was 0.1% formic acid acetonitrile aqueous solution (acetonitrile was 80%). The liquid chromatography column (50 μm × 150 mm, Acclaim PepMapTM RSLC, Thermo scientific Technology Inc., Boston, MA, USA) was equilibrated with 92% of liquid A. The injection volume of 1 μL was separated by the column, and the separation was performed by capillary high-performance liquid chromatography (HPLC), followed by mass spectrometry analysis by a mass spectrometer (Thermo QE HF, Thermo Fisher Scientific Inc., Boston, MA, USA) for mass spectrometry analysis.

The mass spectrometry results were analyzed using an online bioactive peptide prediction tool (https://agbg.shinyapps.io/MultiPep/) (accessed on 21 April 2025).

### 2.20. Statistical Analysis

All the indicators and related data in this experiment were measured in three parallel groups. The data were analyzed, and graphs were plotted using SPSS 26.0 and Origin 2022 software (Chicago, IL, USA), respectively. One way analysis of variance (ANOVA) followed by Tukey post hoc test was performed to evaluate statistical significance, and differences were considered statistically significant at *p* < 0.05.

## 3. Results

### 3.1. Nutritional Components

Table 1 shows the nutritional composition of yak, cattle, and donkey skins. The moisture content ranged from 61.62 to 63.11 g/100 g, with no significant differences observed among samples; HA-YS showed the highest moisture level, while LA-YS had the lowest. However, the protein content of LA-YS was higher than that of other cowhides from different altitudes and breeds, with an increase ranging from 0.41% to 4.49%. In terms of fat content, LA-YS exhibited higher levels than HA-YS (1.64%), PLRS (3.83%), and XJBS (5.46%), but lower than that of DS (4.91%). These findings indicate that cowhides and donkey skin from different sources differ in their nutritional composition, providing a scientific basis for selecting appropriate raw materials for processing and utilization.

### 3.2. Amino Acid

#### 3.2.1. Amino Acid Content

The amino acid composition of yak, cattle, and donkey skins was analyzed, and the results are presented in Table 2. A total of 17 amino acids were identified, among which glycine was the most abundant (5.105–7.14 g/100 g), followed by proline (2.89–4.12 g/100 g) and alanine (2.15–2.82 g/100 g). These amino acids are also the predominant components of collagen, suggesting that cowhides contain a high proportion of collagen protein [28]. Among the samples, the LA-YS exhibited the highest total amino acid content, which significantly higher than that of different altitudes and breeds of hides (6.77–24.63%) (*p* < 0.05). XJBS ranked second, with a total amino acid content significantly higher than that of MA-YS (21.07%), HA-YS (11.43%), and PLRS (9.39%) (*p* < 0.05), while the difference with DS was not statistically significant. Furthermore, LA-YS also showed the highest content of essential amino acids, being significantly higher by 4.91% to 16.32% compared with other samples. XJBS ranked second and was significantly higher than DS (2.69%), MA-YS (19.42%), HA-YS (11.19%), and PLRS (9.29%) (*p* < 0.05). These results indicate significant differences in amino acid content yak, cattle, and donkey skins, highlighting the nutritional diversity and processing potential of cowhides from different sources, and providing a theoretical basis for their evaluation as sources of animal-derived protein.

#### 3.2.2. Flavoring Amino Acids

**Figure 2:**
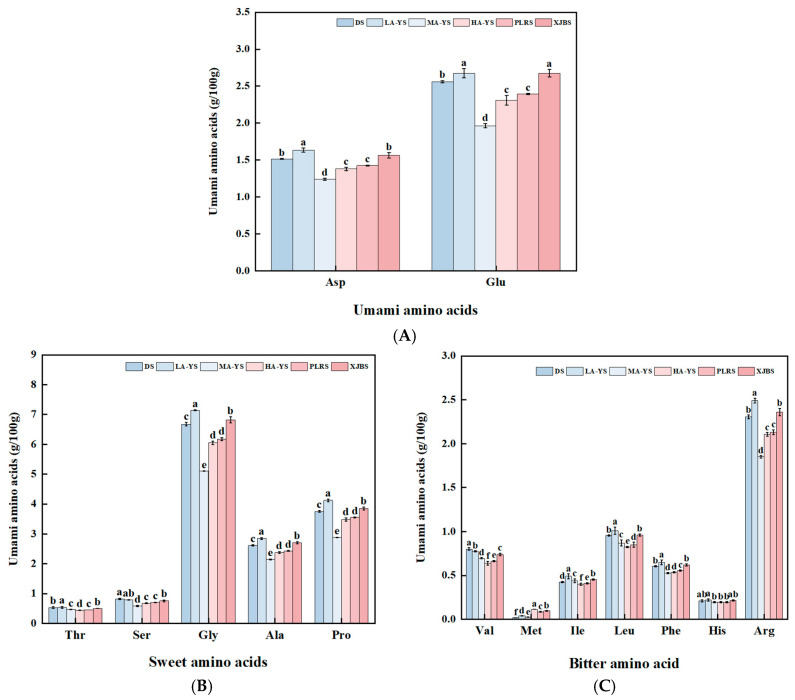
Analysis of flavor amino acids in yak, cow and donkey skins. (**A**) Umami amino acid content. (**B**) Sweet amino acid content. (**C**) Bitter amino acid content. Different lowercase letters indicate *p* < 0.05, meaning there are significant differences among different samples.

As shown in Figure 2A–C and Table 3, all samples primarily contained glutamic acid as the dominant umami amino acid, glycine and proline as the main sweet-tasting amino acids, and cysteine and histidine as the principal bitter-tasting amino acids [29]. LA-YS exhibited the highest content of umami amino acids, showing no significant difference from XJBS, but significantly exceeding the other four samples by 6.96% to 25.52% (*p* < 0.05). Notably, the sweet-tasting amino acids (15.43 g/100 g) and bitter-tasting amino acids (5.67 g/100 g) in LA-YS also showed significant differences compared to all other samples. Overall, both LA-YS and XJBS demonstrated superior profiles in terms of flavor-related amino acid content. Although significant quantitative differences were observed among the samples, the relative proportions of umami, sweet, and bitter amino acids remained relatively consistent. These variations in flavor amino acid content reveal the potential value of cattle and donkey skins from different sources in terms of flavor characteristics and product development for processing applications.

#### 3.2.3. Amino Acid Clustering Heat Map

**Figure 3:**
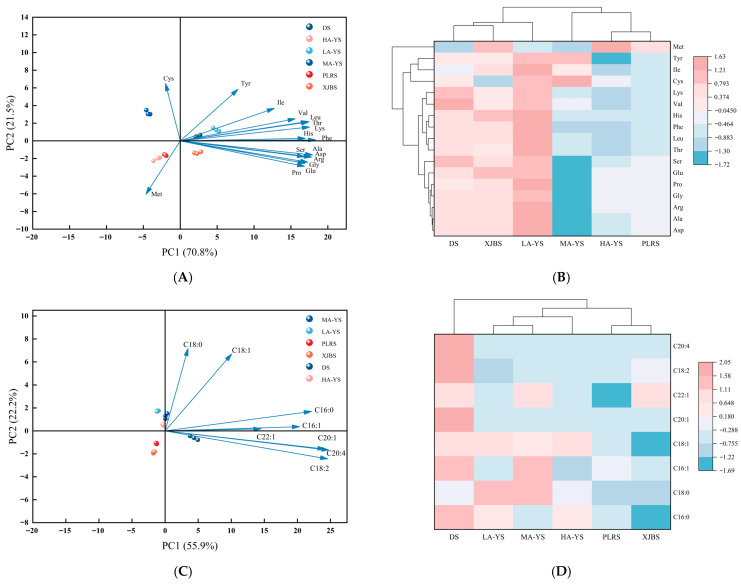
Analysis of amino acids and fatty acids in yak, cow and donkey skins. (**A**,**B**) Amino acid principal component analysis diagram and heat map with dendrogram. (**C**,**D**) Fatty acid principal component analysis diagram and heat map with dendrogram. All values were the means ± SD (n = 3).

Cluster analysis in Figure 3A revealed that, despite differences in lysine and proline content, the amino acid profiles of XJBS and MA-YS were highly similar to that of DS, suggesting their potential as alternative sources. Significant variations in amino acid content were observed among the samples, likely influenced by both breed and altitude. This conclusion was further supported by the principal component analysis (PCA) shown in Figure 3B, where the first two principal components, PC1 (70.8%) and PC2 (21.5%), together accounted for 92.3% of the total variance, indicating a strong explanatory power for the observed differences. DS was clearly separated from cowhides along the PC1 axis, while certain cowhides such as LA-YS and PLRS clustered closely with DS, suggesting compositional similarities. Notably, some samples, such as HA-YS, formed distinct clusters within the PCA space, highlighting the diversity among cowhides and their potential for differentiated functional applications. Overall, these findings indicate that certain cowhides exhibit amino acid profiles comparable to donkey skin, underscoring their potential as alternative collagen sources. Moreover, the observed compositional diversity among cowhides emphasizes their value in tailored functional applications and provides a scientific foundation for future industrial utilization and resource optimization.

### 3.3. Fatty Acid

#### 3.3.1. Fatty Acid Content

The fatty acid composition of six skin-derived samples was analyzed, and the results are shown in Table 4. Significant differences were observed in the total fatty acid content among yak, cattle, and donkey skins. The yak skins exhibited a higher overall total fatty acid content (0.19–0.21 g/100 g), which was significantly greater than that of cowhides (PLRS and XJBS) by 28.57 to 42.11%, but lower than that of DS by 19.23% to 33.33% (*p* < 0.05). A similar trend was found in the monounsaturated fatty acid (MUFA) content, where the yak skins showed 27.27% to 45.45% higher levels than PLRS and XJBS, but were still 15.38 to 23.07% lower than DS (*p* < 0.05). Although the total fatty acid content in yak skins was relatively high, the polyunsaturated fatty acid (PUFA) content in PLRS and XJBS was generally higher than that in the yak samples, exceeding them by 14.28% to 57.14%. Notably, a decreasing trend in fatty acid content was observed with increasing altitude, which may be attributed to environmental factors associated with elevation. Additionally, a total of eight fatty acids were identified in DS, with arachidonic acid and eicosenoic acid detected exclusively in this sample and not in any cowhides, indicating a higher lipid diversity in DS [30]. These findings reveal substantial differences in the fatty acid composition of yak, cattle, and donkey skins, providing a scientific basis for evaluating their nutritional characteristics and potential applications in industry.

#### 3.3.2. Fatty Acid Clustering Heat Map

Figure 3C,D present the results of principal component analysis (PCA) and hierarchical clustering, providing a systematic comparison of the fatty acid composition among different cattle skin and donkey skin samples. As shown in Figure 3C, PC1 and PC2 together explained 78.1% of the total variance, indicating clear differences in fatty acid profiles among the samples. Stearic acid (C18:0) and oleic acid (C18:1) contributed most significantly to PC1, while eicosenoic acid (C20:1) and arachidonic acid (C20:4) had the greatest influence on PC2, which is consistent with previous reports on breed-specific fatty acid variations [31]. Figure 3D further demonstrates that MA-YS exhibited notably higher levels of palmitoleic acid (C16:1) and erucic acid (C22:1) compared to all samples except DS. In particular, the fatty acid content of yak skins was significantly higher than that of the conventional cowhides (PLRS and XJBS) yet remained significantly lower than that of DS. These compositional differences are likely driven by a combination of environmental factors, altitude, and breed-specific genetic backgrounds. Overall, the PCA and clustering analysis of fatty acid profiles provide a scientific foundation for the selection of specific raw materials based on lipid characteristics and the development of value-added products.

### 3.4. SDS-PAGE

**Figure 4:**
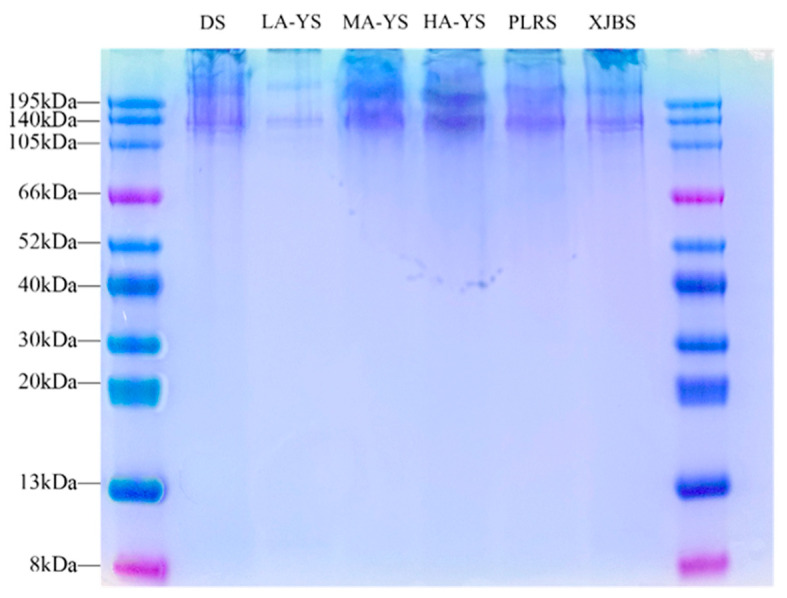
SDS-PAGE analysis image of different collagen samples.

SDS-PAGE is a widely used method for assessing the purity, molecular composition, and aggregation state of collagen extracts. It effectively reveals the presence of α-chains, β/γ-polymers, and potential cross-linked byproducts [31]. As shown in the SDS-PAGE profiles (Figure 4), collagen samples from yak, cattle, and donkey skins exhibited minimal bands around 195 kDa, indicating negligible residual aggregates or cross-linked byproducts and suggesting high extraction purity. All samples displayed two closely aligned bands at approximately 130 kDa, corresponding to the α1 and α2 chains of type I collagen, confirming the structural integrity of the collagen molecules [32].

In addition, no prominent bands were detected below 66 kDa, suggesting that the collagen was primarily composed of monomeric α-chains, with low levels of β- and γ-chain dimers and trimers. Notably, MA-YS and HA-YS exhibited stronger band intensity around 140 kDa, indicating the presence of more stable triple-helical structures, consistent with their superior thermal stability [33]. Collectively, the SDS-PAGE results not only confirm the high purity and structural integrity of the extracted collagens but also highlight molecular stability differences among samples, reflecting potential structure–function relationships.

### 3.5. Thermal Stability

**Figure 5:**
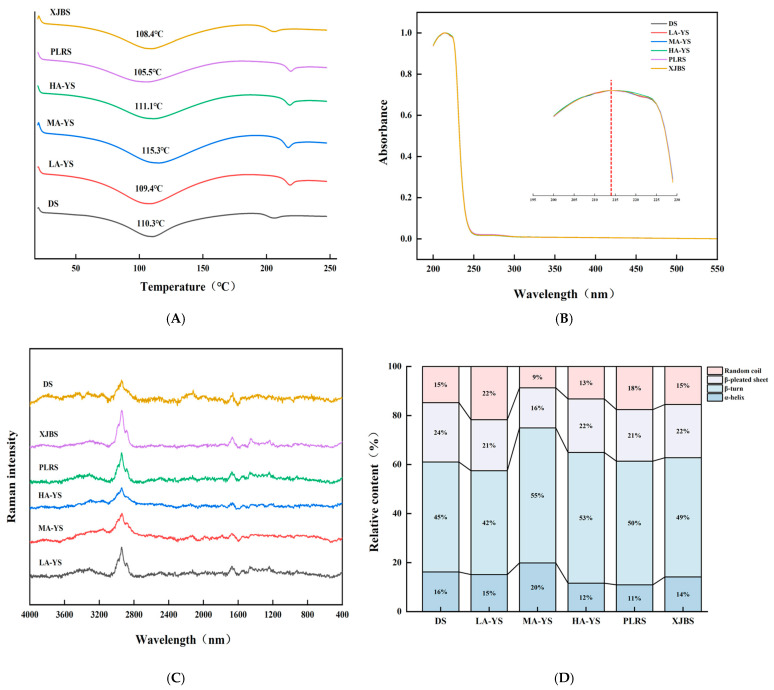
Comparison of yak, cattle, and donkey skins collagen structures. (**A**) Ultraviolet spectral absorption diagram. (**B**) Comparison of collagen thermal stability. Raman spectra and percentage diagrams of secondary structures of different collagens (**C**,**D**).

Differential scanning calorimetry (DSC) was employed to evaluate the thermal stability of collagen. As shown in Figure 5A, excluding DS, yak skin samples exhibited generally higher thermal stability than cattle skin samples (PLRS and XJBS). The denaturation temperature of yak skin collagen is 109.4–115.3 °C, which is consistent with the previous research results of yak skin gelatin (112.37 °C) [34]. Among the samples, HA-YS showed the highest denaturation temperature (115.3 °C), which may be attributed to its higher degree of molecular cross-linking. This is consistent with the findings of Pal et al. [35], who reported a strong correlation between collagen cross-linking and thermal stability. The morphological characteristics observed by scanning electron microscopy in this study further support this conclusion. These results indicate that collagen from yak skin, especially HA-YS, may be more suitable for high-temperature applications.

### 3.6. Structural Properties

#### 3.6.1. Raman Spectroscopy

The secondary structure of collagen mainly includes α-helix, β-sheet, β-turn, and random coil forms, among which the α-helix structure is crucial for maintaining the stability of its triple helix and is generally associated with thermal stability and mechanical strength [36]. As shown in Figure 5C,D, MA-YS collagen exhibited the highest α-helix content (20%), which is related to its enhanced thermal stability. Zheng et al. [27] found that the higher the proportion of α-helix in fish skin collagen, the higher its denaturation temperature.

The β-sheet structure contributes to the mechanical support of collagen fibers; an increase in random coil content may enhance protein chain flexibility and promote the exposure of hydrophobic groups, thereby improving interfacial activity [37]. LA-YS collagen contained relatively high proportions of β-sheet and random coil structures (43%), which may facilitate hydrophobic group exposure and thus improve its emulsifying ability, Chen et al. [38] reported that ultrasound treatment increased the content of disordered structures (β-sheet and random coil) in pea protein, exposing more hydrophobic groups and enhancing emulsifying activity. Similarly, DS collagen showed 16% α-helix and 39% β-sheet and random coil, which also contributed to its good stability and high emulsifying activity. These structural differences collectively emphasize that the secondary structure composition of collagen from different altitudes and breeds partially determines its functional properties and application potential, providing a basis for selecting collagen sources suitable for processing adaptations.

#### 3.6.2. Ultraviolet Scanning Spectrum

Collagen contains chromophoric groups such as carboxyl (COOH), amide (CONH_2_), and carbonyl (C=O), which exhibit a prominent absorption peak near 220 nm. As it contains merely minor quantities of aromatic amino acids such as phenylalanine and tyrosine, a pronounced absorption peak at approximately 280 nm is absent. Ultraviolet absorption spectra represent the cumulative contribution of various ultraviolet chromophores within protein molecules and serve as important indicators for collagen type identification [39]. As shown in Figure 5B, collagen from DS, MA-YS, HA-YS, and PLRS reached their absorption maxima at 214 nm, while LA-YS and XJBS peaked at 215 nm. Moreover, no absorption peaks were observed at 280 nm across all collagen samples from different altitudes and breeds, indicating high purity of the extracted collagen with minimal contamination from non-collagen proteins. These observations are consistent with the research results of Zhu et al. [8]. Collagen exhibited a characteristic peak near 220 nm but did not show a characteristic peak at 280 nm, which is the result of the n-π* transition of the carbonyl group in the β-chain of collagen. The ultraviolet absorption peak positions of collagen from various sources provide distinctive spectral features that conform to the established ultraviolet absorption patterns of collagen, thereby offering a reliable basis for its identification and analysis.

### 3.7. SEM

**Figure 6:**
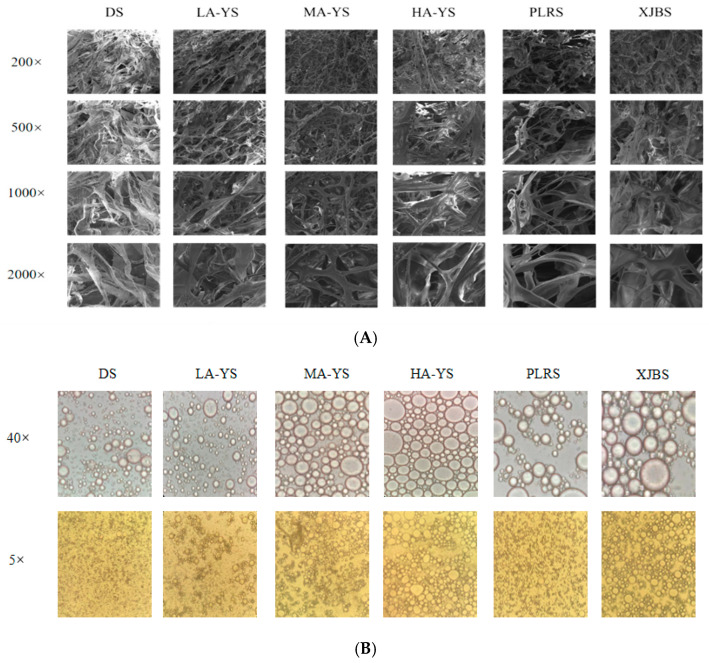
Yak, cattle, and donkey skins collagen microstructures. (**A**) Collagen structure under scanning electron microscope. (**B**) Emulsion state under optical microscope.

Scanning electron microscopy (SEM) observations of the extracted collagen revealed that all samples exhibited irregular porous network structures on their surfaces, a characteristic commonly observed in purified collagen under natural conditions [40], consistent with the findings of this study. As shown in Figure 6A, MA-YS collagen fibers were finer, more uniform, and had smooth and intact edges, indicating minimal structural damage during processing and correlating with its superior thermal stability as confirmed by the results in Figure 5A. In contrast, PLRS and XJBS collagen fibers appeared coarser with rough and indistinct edges, occasionally exhibiting irregular entanglements, suggesting a tendency to fracture under applied stress These differences likely arise from variations in intermolecular hydrogen bonding interactions [19].Therefore, MA-YS collagen is particularly suitable for high-precision, structure-sensitive applications, whereas PLRS and XJBS collagen may require additional stabilization to meet the demands of more rigorous applications.

### 3.8. Emulsifying Properties

**Figure 7:**
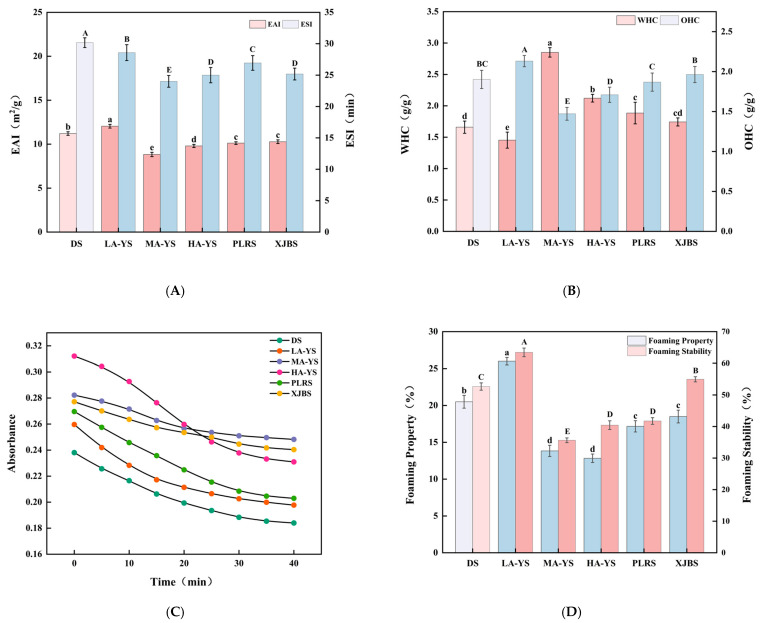
Functionality of yak, cattle, and donkey skins collagen emulsions. (**A**) Emulsifying properties. (**B**) Water and oil holding capacity. (**C**) Turbidity changes from 0 to 40 min. (**D**) Foaming properties and foaming stability. Different letters indicate significant differences (*p* < 0.05). All values were the means ± SD (n = 3).

Emulsifying properties, as a critical processing characteristic of collagen, not only influence its dispersion and stability within composite food systems but also directly relate to its interfacial conformation and structural features. Ruan et al. [41] demonstrated that molecular structure, amino acid composition, and the distribution of hydrophobic groups in collagen significantly affect both emulsifying activity and emulsifying stability, with these factors collectively determining the emulsification performance of collagen from different breeds. Figure 7A presents the emulsifying activity index (EAI) and emulsifying stability index (ESI) of collagen extracted from skins of various breeds and altitudes. Among these, LA-YS exhibited the highest EAI (12.05 m^2^/g), significantly surpassing the other five samples by 6.94% to 26.74%, indicating stronger interfacial activity likely related to the distribution of hydrophobic groups within its protein structure [42]. Notably, the emulsifying activity of yak collagen decreased with increasing altitude, presumably due to subtle structural alterations induced by altitude that affect its adsorption capacity at the emulsion interface.

Conversely, DS collagen demonstrated the most outstanding emulsifying stability (30.17 min), significantly exceeding all other samples by 5.25% to 20.41% (*p* < 0.05). This superior stability may result from the formation of a denser and more stable collagen network at the oil–water interface, which inhibits droplet aggregation and enhances the emulsion system’s overall stability [43]. In comparison, the emulsifying properties of MA-YS and HA-YS collagen were poorer relative to cowhides (PLRS and XJRS), suggesting that emulsions formed by these samples are more prone to creaming or phase separation during storage. These differences in emulsification capacity among collagen sources reflect the coupled effects of their structural characteristics and interfacial behavior, providing a theoretical basis for the targeted application of collagen in specific food emulsion systems.

### 3.9. Electron Microscopes

Optical microscopy effectively reveals the microstructure and droplet morphology of collagen emulsions, demonstrating their ability to adsorb at the oil–water interface and stabilize droplets during emulsification [44]. Comparative optical microscopy images show that emulsions from all samples exhibit spherical structures, indicating collagen adsorption at the oil–water interface, thereby stabilizing the droplets. At 40× magnification (Figure 6B), all collagen emulsions form regular spherical droplets; notably, DS collagen produces smaller and more compact droplets compared to other samples, demonstrating its superior ability to adsorb and stabilize droplets at the interface, resulting in higher emulsion stability [45]. However, at 5× magnification, LA-YS collagen emulsions display smaller average droplet sizes and better dispersibility, suggesting faster interfacial adsorption rates and higher emulsifying activity. It is also noteworthy that collagen emulsions from cattle skins (PLRS and XJBS) exhibit relatively uniform droplets, indicating good emulsifying performance. Overall, the droplet size and distribution characteristics revealed by microscopy are highly consistent with the emulsification data presented in Figure 7A, further corroborating the differences in emulsion formation among collagens from yak, cattle, and donkey skins.

### 3.10. Water Holding and Oil Holding Properties

**Figure 8:**
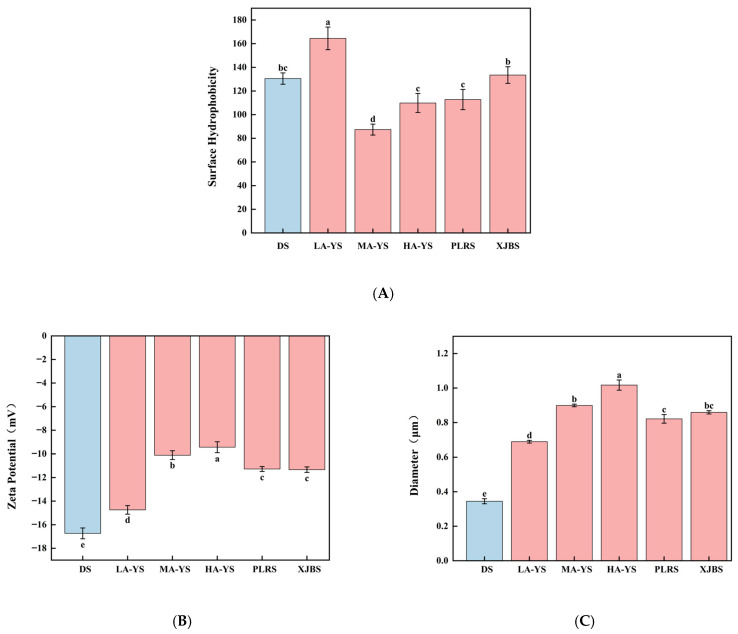
Yak, cattle, and donkey skins collagen stability. (**A**) Surface hydrophobicity. (**B**) Zeta potential value. (**C**) Particle size. Different letters indicate significant differences (*p* < 0.05). All values were the means ± SD (n = 3).

Water-holding capacity (WHC) and oil-holding capacity (OHC) are crucial indicators of the hydrophilic–hydrophobic balance and structural characteristics of collagen, widely used to assess its functional potential in various food systems. As shown in Figure 7B, a systematic comparison of WHC and OHC revealed differences in the functional properties among the three types of collagens, which are crucial for enhancing water-binding capacity. Among all samples, MA-YS exhibited the highest WHC (2.85 g/g), which was higher than that of sour cherry kernel concentrate protein (2.42 g/g) [46] and chicken foot collagen (1.9 g/g) [47], suggesting the presence of more hydrophilic groups in its molecular structure. In contrast, LA-YS had the lowest WHC (1.45 g/g), likely due to its higher content of hydrophobic amino acids (Table 2) and stronger surface hydrophobicity (Figure 8A), which restricts its water-binding capacity [48].

Notably, although LA-YS collagen exhibited poor WHC, its OHC was significantly higher than that of the other five samples by 7.89–30.96% (*p* < 0.05), indicating excellent lipid-binding ability. Previous studies have shown that OHC is not only influenced by the hydrophobic amino acid content but also closely related to the degree of molecular curling [49]. The higher proportion of nonpolar amino acids (e.g., Val, Leu, Phe) in LA-YS may enhance hydrophobic interactions between the protein and lipids, thereby improving its oil-binding ability. In comparison, PLRS and XJBS exhibited higher OHC than MA-YS and HA-YS, but still lower than LA-YS, suggesting that cattle skin collagen has potential in lipid adsorption, although its effectiveness may be limited by differences in molecular flexibility or spatial conformation that reduce the exposure of lipid-binding sites. These variations in WHC and OHC among collagens from different altitudes and breeds reflect their structural diversity, providing theoretical support for their functional applications in specific food systems. These research results indicate that environmental factors related to altitude (such as temperature and oxygen content) may indirectly affect the hydrophilic-hydrophobic balance and molecular organization of collagen in yak skin, thereby influencing its WHC and OHC.

### 3.11. Surface Hydrophobicity

The emulsification capacity of a protein system is closely associated with its surface hydrophobicity, as the degree of exposed hydrophobic groups directly influences its ability to adsorb at the oil–water interface and stabilize emulsion droplets [49]. As shown in Figure 8A, LA-YS collagen exhibited the highest surface hydrophobicity (163.12 μg), significantly greater than that of the other five collagens by 18.86–46.89% (*p* < 0.05), indicating a greater number of exposed hydrophobic groups. This structural trait facilitates rapid interfacial adsorption, thereby enhancing emulsifying activity [50], which is consistent with the higher EAI observed for LA-YS in Figure 7A. In contrast, MA-YS collagen showed the lowest surface hydrophobicity, suggesting limited exposure of hydrophobic residues, which may be due to a more compact molecular conformation or burial of hydrophobic side chains within the protein interior. This structural feature favors the full exposure of hydrophilic groups on the molecular surface, thus strengthening the interaction between collagen and water molecules, as evidenced by its superior water-holding capacity (2.85 g/g, Figure 7B). Therefore, the excellent WHC of MA-YS collagen may be attributed to its stronger surface hydrophilicity. Overall, the variation in surface hydrophobicity among collagens from yak, cattle, and donkey skins can be ascribed to differences in hydrophobic amino acid composition and spatial conformation. These findings further highlight the relationship between collagen’s structural features and its functional diversity.

### 3.12. Particle Size, Zeta Potential

Zeta potential and emulsion droplet size are critical physical indicators for evaluating the emulsification stability of collagen, as they reflect the balance of electrostatic repulsion and dispersion stability among particles [51]. As shown in Figure 8B, DS collagen exhibited the highest Zeta potential (16.73 mV), significantly higher than that of all cowhide collagens by 11.93–43.58% (*p* < 0.05), indicating stronger electrostatic repulsion between molecules. This favors the formation of a more stable dispersion structure in emulsified systems. Xu et al. [52] also reported that increased surface negative charge of collagen enhances colloidal stability, thereby improving overall emulsion stability.

Figure 8C further illustrates the average droplet size variation among the collagen emulsions, with all samples ranging from 0.3448 μm to 1.0171 μm. Notably, DS and LA-YS collagen emulsions exhibited relatively smaller droplet sizes, suggesting a more efficient dispersion of the oil phase during emulsification and the formation of a denser interfacial structure, which contributes to greater emulsion stability. These findings are consistent with microscopic observations of droplet morphology and align with the study by Ma et al. [53], which emphasized that smaller droplet sizes contribute to enhanced physical stability and functional performance of emulsions.

### 3.13. Turbidity

Figure 7C illustrates the turbidity variations in different collagen emulsions over a 0–40 min period, providing insight into droplet aggregation behavior and interfacial stability. At the initial time point (0 min), MA-YS exhibited the highest turbidity (0.309), suggesting the formation of larger droplet aggregates during the early dispersion stage. This may be attributed to its lower Zeta potential, which weakens the electrostatic repulsion between molecules. In contrast, DS collagen showed the lowest initial turbidity, indicating a more stable dispersion state and stronger anti-aggregation ability. This observation aligns with its highest Zeta potential recorded in Figure 8B, corroborating the role of intermolecular electrostatic repulsion in enhancing emulsion stability. Yang et al. [54] reported that when the absolute value of the Zeta potential of soy protein isolate decreases, its solubility declines and aggregation increases, resulting in higher turbidity.

Figure 7C further highlights the turbidity changes across 0–40 min, evaluating the emulsions’ dispersion and aggregation trends during static storage. DS and LA-YS collagen emulsions, which exhibited smaller droplet sizes (Figure 8C), presented relatively lower initial turbidity values, indicating more uniform and compact droplet structures and better initial dispersion. However, LA-YS showed a greater turbidity decline over time than DS, suggesting that its interfacial membrane is less compact, with a higher risk of droplet coalescence or flocculation, ultimately leading to lower emulsion stability. It is also worth noting that MA-YS collagen exhibited the highest initial turbidity (0.309), reflecting the presence of larger and more heterogeneously distributed droplets, consistent with its largest average droplet size (1.0171 μm) shown in Figure 8C. Taken together, the turbidity variations among these collagen emulsions were jointly influenced by droplet size and surface charge characteristics, highlighting the structural determinants underlying their differences in emulsifying stability.

### 3.14. Foaming Properties and Foaming Stability

Foaming capacity and foam stability are key parameters for evaluating the interfacial activity of collagen, as they reflect its ability to adsorb at the air–liquid interface, expand, and stabilize the foam structure-features that are of considerable significance for the development of structurally designed food products [55]. As shown in Figure 7D, LA-YS collagen exhibited the highest foaming capacity (26%), which was significantly greater than that of other cowhide collagen samples by 21.15% to 50.64% (*p* < 0.05), indicating its superior interfacial expansion and foam-forming capabilities. This is consistent with the foaming property found in low-molecular-weight yak skin gelatin [56]. This performance may be attributed to the greater exposure of hydrophobic groups in its molecular structure, facilitating rapid adsorption at the air–liquid interface and efficient foam generation, which is also consistent with its high surface hydrophobicity (Figure 8A).

In terms of foam stability, LA-YS again showed the best performance, surpassing the other samples by 13.36% to 43.82% (*p* < 0.05), suggesting that it can quickly form a dense interfacial film after foaming, thereby slowing liquid drainage and preventing bubble rupture, resulting in enhanced foam stability [57]. In contrast, MA-YS collagen exhibited relatively poor foaming capacity and foam stability, implying a dual disadvantage in foam film formation and stabilization, which may result from its lower exposure of hydrophobic moieties, thereby limiting its interfacial activity. Collectively, LA-YS collagen demonstrated synergistic advantages in both foam formation and stabilization, indicating its excellent interfacial functionality and promising potential for application in food systems that require controlled foam structures.

### 3.15. Peptides Prediction

**Figure 9:**
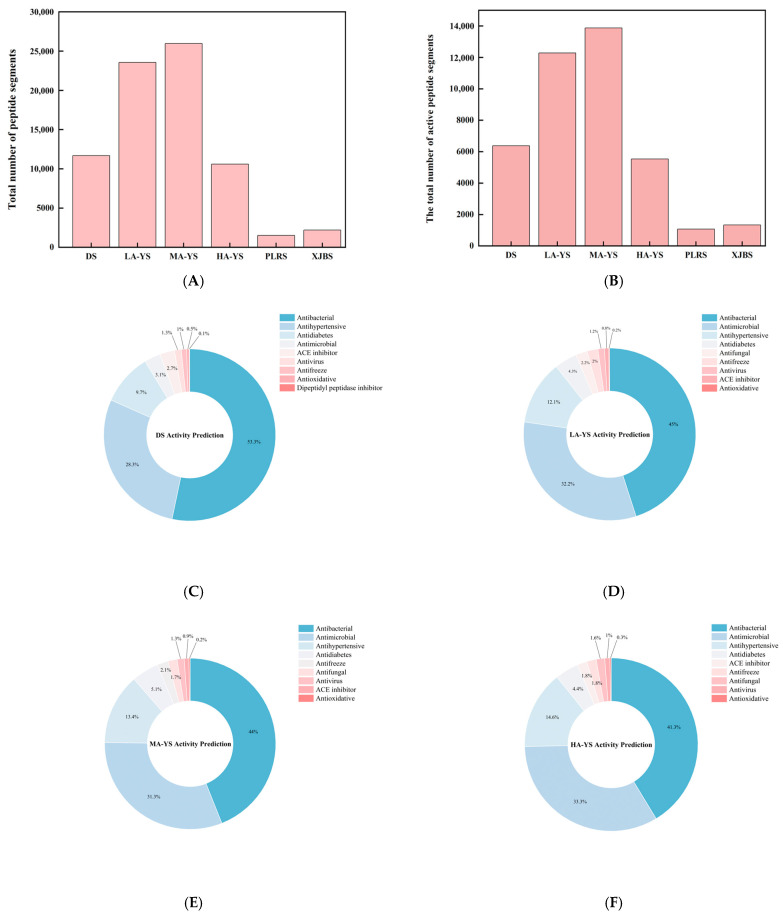

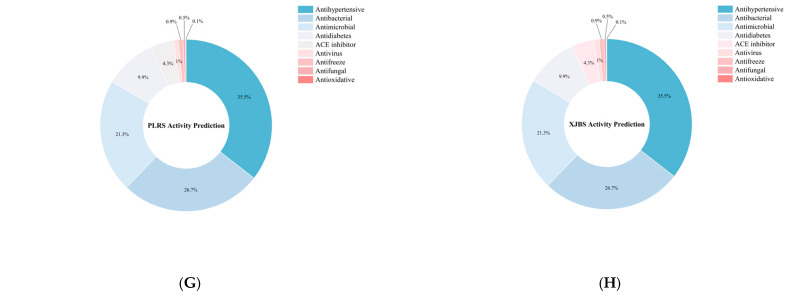
Identification results of yak, cattle, and donkey skins active peptides. (**A**) Total number of peptides segments in cowhide and donkey hide. (**B**) Number of active peptide segments in cowhide and donkey hide. (**C**–**H**) (DS, LA-YS, MA-YS, HA-YS, PLRS, XJBS) Types of active peptides and proportions of inhibitory active peptides.

Protein fragmentation, theoretical peptide matching, and identification were conducted using Proteome Discoverer 2.5, with protein annotation performed based on the corresponding species-specific protein databases. The resulting database search provided both protein and matched peptide information, which was subsequently subjected to bioactivity prediction using dedicated online tools. The study systematically analyzed the composition and functional classification of potential bioactive peptides from yak, cattle, and donkey skins. As shown in Figure 9A, yak skin samples LA-YS, MA-YS, and HA-YS contained 23,558, 25,966, and 10,599 peptides, respectively, substantially higher than those identified in donkey skin (11,678 peptides) and cowhides (PLRS: 1515 peptides; XJBS: 2186 peptides), indicating yak skin as a rich reservoir of potential functional peptides.

As shown in Figure 9B, the distribution of bioactive peptides varied significantly among different hide sources. Further analysis showed (Figure 9C–H) that yak skins exhibited higher levels of antibacterial peptides (27.47–29.75%), while Pingliang red cattle and Xinjiang brown cattle skins were enriched in peptides with antihypertensive potential. In contrast, donkey skin collagen showed superior antimicrobial activity, indicating distinct functional advantages associated with each species. Although donkey skin showed a slightly higher proportion of bioactive peptides (54.7%), yak samples maintained consistently high bioactivity ratios (>52%), demonstrating both peptide richness and functional diversity. In contrast, while the relative proportion of bioactive peptides in PLRS and XJBS was higher (70.56% and 61.07%, respectively), their total peptide counts were much lower, suggesting that cowhides have limited peptide abundance and structural diversity, thus restricting their potential for bioactive peptide development.

The high abundance and structural variety of bioactive peptides in yak skins highlight its promising potential for applications in functional foods, nutraceuticals, and peptide-based therapeutics. In particular, the richness in antihypertensive, antimicrobial, antidiabetic, and neuromodulatory peptides provides a strong functional basis for dietary interventions in chronic disease management. Its well-balanced bioactivity profile also supports its suitability for incorporation into multifunctional food systems such as health beverages and peptide-enriched supplements. Future studies should focus on the isolation and validation of key functional peptide sequences and elucidate their mechanisms of action through both in vitro and in vivo models.

## 4. Conclusions

This study systematically analyzed the differences in nutritional indicators, collagen physicochemical and functional properties, and peptide bioactivities among yak, cattle, and donkey skins. The results showed that altitude significantly affected the structural and functional characteristics of yak skin collagen: MA YS exhibited higher water holding capacity and thermal stability, whereas LA YS showed superior lipid binding capacity and foaming ability, attributed to its smaller particle size and greater surface hydrophobicity. These structural traits directly enhanced its interfacial expansion and adsorption performance. Yak skin collagen also contained abundant bioactive peptides with strong antidiabetic and antibacterial potential. Across species, donkey skin collagen exhibited the highest zeta potential and emulsion stability, with minimal turbidity variation within 40 min, indicating excellent dispersion stability. Overall, yak skin collagen demonstrated balanced structural integrity, outstanding emulsifying and foaming capacities, and rich bioactive peptide profiles, highlighting a strong structure function correlation. These features suggest its high potential as a functional ingredient in collagen-based emulsions, gels, and encapsulation systems, supporting its industrial and nutritional valorization. Nevertheless, large-scale application still faces challenges related to extraction efficiency, production cost, and process standardization. Furthermore, sustainable sourcing of animal skins and environmental considerations in collagen extraction and purification should be carefully addressed to ensure the long-term feasibility of industrial utilization.

## Figures and Tables

**Table 1:** The nutritional composition of yak, cattle and donkey skins.

Content (g/100 g)	Variety					
	DS	LA-YS	MA-YS	HA-YS	PLRS	XJBS
Moisture	61.68 ± 0.34 ^a^	61.62 ± 0.06 ^a^	62.74 ± 0.02 ^a^	63.11 ± 0.12 ^a^	62.98 ± 0.10 ^a^	61.78 ± 0.06 ^a^
Protein	35.28 ± 0.48 ^a^	35.58 ± 0.65 ^a^	33.98 ± 0.44 ^b^	34.15 ± 0.67 ^b^	34.38 ± 1.84 ^b^	35.43 ± 1.13 ^a^
Fat	1.92 ± 0.14 ^a^	1.83 ± 0.07 ^b^	1.80 ± 0.03 ^b^	1.78 ± 0.11 ^c^	1.76 ± 0.06 ^c^	1.73 ± 0.16 ^d^
Ash	0.27 ± 0.002 ^a^	0.24 ± 0.005 ^c^	0.24 ± 0.019 ^c^	0.24 ± 0.003 ^c^	0.26 ± 0.008 ^ab^	0.25 ± 0.003 ^bc^

The results are expressed as the mean ± standard error (n = 3), and different letters in the same row indicate significant differences between samples (*p* < 0.05). DS: Donkey skin, LA-YS: Low-altitude yak skin, MA-YS: Medium-altitude yak skin, HA-YS: High-altitude yak skin, PLRS: Pingliang red cattle skin, XJBS: Xinjiang brown cattle skin.

**Table 2:** Amino acid content of yak, cattle, and donkey skins.

Amino Acids (g/100 g)	Variety					
	DS	LA-YS	MA-YS	HA-YS	PLRS	XJBS
Thr	0.510 ± 0.000 ^b^	0.560 ± 0.000 ^a^	0.460 ± 0.000 ^d^	0.445 ± 0.007 ^c^	0.460 ± 0.000 ^c^	0.505 ± 0.007 ^b^
Val	0.800 ± 0.014 ^a^	0.775 ± 0.007 ^b^	0.695 ± 0.007 ^d^	0.640 ± 0.000 ^f^	0.665 ± 0.007 ^e^	0.740 ± 0.014 ^c^
Met	0.018 ± 0.001 ^f^	0.039 ± 0.007 ^d^	0.026 ± 0.001 ^e^	0.115 ± 0.001 ^a^	0.086 ± 0.003 ^c^	0.096 ± 0.004 ^b^
Leu	0.955 ± 0.007 ^b^	1.010 ± 0.000 ^a^	0.870 ± 0.000 ^c^	0.825 ± 0.007 ^e^	0.850 ± 0.000 ^d^	0.960 ± 0.014 ^b^
IIe	0.425 ± 0.007 ^d^	0.490 ± 0.000 ^a^	0.440 ± 0.000 ^c^	0.400 ± 0.000 ^f^	0.410 ± 0.000 ^e^	0.455 ± 0.007 ^b^
Phe	0.605 ± 0.007 ^b^	0.650 ± 0.000 ^a^	0.525 ± 0.007 ^d^	0.535 ± 0.007 ^d^	0.555 ± 0.007 ^c^	0.620 ± 0.014 ^b^
Lys	1.120 ± 0.000 ^a^	1.130 ± 0.014 ^a^	0.945 ± 0.007 ^c^	0.935 ± 0.021 ^c^	0.945 ± 0.007 ^c^	1.050 ± 0.014 ^b^
TEAA	4.433 ± 0.020 ^b^	4.655 ± 0.021 ^a^	3.961 ± 0.005 ^c^	3.895 ± 0.043 ^c^	3.97 ± 0.004 ^c^	4.426 ± 0.075 ^b^
Asp	1.515 ± 0.007 ^b^	1.635 ± 0.021 ^a^	1.240 ± 0.028 ^d^	1.380 ± 0.014 ^c^	1.425 ± 0.007 ^c^	1.565 ± 0.035 ^b^
Ser	0.810 ± 0.000 ^a^	0.790 ± 0.014 ^b^	0.585 ± 0.021 ^e^	0.670 ± 0.000 ^f^	0.705 ± 0.007 ^d^	0.760 ± 0.028 ^c^
Glu	2.560 ± 0.000 ^b^	2.675 ± 0.064 ^a^	1.965 ± 0.063 ^d^	2.310 ± 0.028 ^c^	2.395 ± 0.007 ^c^	2.675 ± 0.049 ^a^
Gly	6.670 ± 0.057 ^c^	7.140 ± 0.014 ^a^	5.105 ± 0.007 ^e^	6.050 ± 0.057 ^d^	6.175 ± 0.049 ^d^	6.820 ± 0.099 ^b^
Ala	2.615 ± 0.021 ^c^	2.820 ± 0.000 ^a^	2.150 ± 0.014 ^e^	2.380 ± 0.028 ^d^	2.430 ± 0.014 ^d^	2.705 ± 0.035 ^b^
Cys	0.027 ± 0.002 ^b^	0.035 ± 0.004 ^a^	0.042 ± 0.004 ^a^	0.022 ± 0.001 ^bc^	0.018 ± 0.001 ^c^	0.015 ± 0.001 ^c^
Tyr	0.205 ± 0.007 ^b^	0.220 ± 0.000 ^a^	0.220 ± 0.000 ^a^	0.185 ± 0.007 ^c^	0.195 ± 0.007 ^bc^	0.205 ± 0.007 ^b^
His	0.210 ± 0.000 ^ab^	0.220 ± 0.014 ^a^	0.195 ± 0.007 ^b^	0.195 ± 0.007 ^b^	0.195 ± 0.007 ^b^	0.215 ± 0.007 ^ab^
Arg	2.305 ± 0.021 ^b^	2.490 ± 0.000 ^a^	1.850 ± 0.014 ^d^	2.105 ± 0.021 ^c^	2.13 ± 0.028 ^c^	2.360 ± 0.042 ^b^
Pro	3.750 ± 0.028 ^c^	4.120 ± 0.042 ^a^	2.885 ± 0.007 ^e^	3.475 ± 0.049 ^d^	3.55 ± 0.014 ^d^	3.845 ± 0.049 ^b^
NEAA	20.667 ± 0.092 ^c^	22.145 ± 0.117 ^a^	16.2365 ± 0.062 ^e^	18.7715 ± 0.151 ^d^	19.218 ± 0.091 ^d^	21.165 ± 0.239 ^b^
HEAA	9.168 ± 0.069 ^c^	9.905 ± 0.05 ^a^	7.591 ± 0.008 ^f^	8.367 ± 0.093 ^e^	8.546 ± 0.025 ^d^	9.421 ± 0.139 ^b^
TAA	25.100 ± 0.149 ^b^	26.800 ± 0.187 ^a^	20.198 ± 0.093 ^d^	22.666 ± 0.256 ^c^	23.189 ± 0.133 ^c^	25.591 ± 0.413 ^b^

The results are expressed as the mean ± standard error (n = 3), and different letters in the same row indicate significant differences between samples (*p* < 0.05). DS: Donkey skin, LA-YS: Low-altitude yak skin, MA-YS: Medium-altitude yak skin, HA-YS: High-altitude yak skin, PLRS: Pingliang red cattle skin, XJBS: Xinjiang brown cattle skin.

**Table 3:** Content of taste amino acids in yak, cattle, and donkey skins.

Variety	Umami Amino Acids		Sweet Amino Acids		Bitter Amino Acid	
	Content (g/100 g)	Total Amino Acid Ratio (%)	Content (g/100 g)	Total Amino Acid Ratio (%)	Content (g/100 g)	Total Amino Acid Ratio (%)
DS	4.01 ± 0.74 ^b^	16.24%	14.36 ± 2.50 ^c^	57.19%	4.89 ± 0.68 ^c^	21.09%
LA-YS	4.31 ± 0.74 ^a^	16.08%	15.43 ± 2.70 ^a^	57.58%	5.67 ± 0.81 ^a^	21.17%
MA-YS	3.21 ± 0.51 ^d^	15.87%	11.19 ± 1.91 ^e^	55.38%	5.32 ± 0.75 ^b^	21.19%
HA-YS	3.69 ± 0.66 ^c^	16.28%	13.02 ± 2.30 ^d^	57.44%	4.60 ± 0.60 ^d^	22.78%
PLRS	3.82 ± 0.69 ^c^	16.47%	14.36 ± 2.34 ^c^	57.44%	4.81 ± 0.67 ^c^	21.24%
XJBS	4.24 ± 0.56 ^a^	16.57%	14.64 ± 2.58 ^b^	57.19%	5.45 ± 0.76 ^b^	21.28%

The results are expressed as the mean ± standard error (n = 3), and different letters in the same row indicate significant differences between samples (*p* < 0.05). DS: Donkey skin, LA-YS: Low-altitude yak skin, MA-YS: Medium-altitude yak skin, HA-YS: High-altitude yak skin, PLRS: Pingliang red cattle skin, XJBS: Xinjiang brown cattle skin.

**Table 4:** Fatty acid content of yak, cattle, and donkey skins.

Fatty Acids (g/100 g)	Variety					
	DS	LA-YS	MA-YS	HA-YS	PLRS	XJBS
C16:0	0.07 ± 0.012 ^a^	0.05 ± 0.044 ^b^	0.04 ± 0.005 ^b^	0.05 ± 0.001 ^b^	0.04 ± 0.010 ^b^	0.02 ± 0.001 ^b^
C18:0	0.03 ± 0.005 ^b^	0.04 ± 0.009 ^a^	0.04 ± 0.005 ^a^	0.03 ± 0.001 ^b^	0.02 ± 0.006 ^c^	0.02 ± 0.001 ^c^
SFA	0.10 ± 0.017 ^a^	0.10 ± 0.053 ^a^	0.08 ± 0.010 ^a^	0.08 ± 0.002 ^a^	0.06 ± 0.015 ^b^	0.04 ± 0.002 ^b^
C16:1	0.01 ± 0.002 ^a^	0.004 ± 0.012^c^	0.01 ± 0.001 ^a^	0.003 ± 0.0001 ^c^	0.006 ± 0.002 ^b^	0.004 ± 0.0003 ^c^
C18:1	0.09 ± 0.016 ^a^	0.09 ± 0.0085 ^a^	0.08 ± 0.008 ^a^	0.09 ± 0.0001 ^a^	0.06 ± 0.016 ^b^	0.04 ± 0.002 ^b^
C20:1	0.006 ± 0.0002	ND	ND	ND	ND	ND
C22:1	0.02 ± 0.003 ^a^	0.01 ± 0.0007 ^bc^	0.02 ± 0.002 ^a^	0.01 ± 0.0004 ^ab^	0.007 ± 0.002 ^c^	0.02 ± 0.001 ^a^
MUFA	0.13 ± 0.021 ^a^	0.11 ± 0.018 ^ab^	0.11 ± 0.011 ^ab^	0.10 ± 0.004 ^b^	0.08 ± 0.020 ^b^	0.06 ± 0.004 ^c^
C18:2	0.02 ± 0.004 ^a^	0.003 ± 0.003 ^c^	0.006 ± 0.0007 ^b^	0.005 ± 0.0001 ^b^	0.006 ± 0.0003 ^b^	0.007 ± 0.0005 ^b^
C20:4	0.007 ± 0.001 ^a^	ND	ND	ND	ND	ND
PUFA	0.03 ± 0.005 ^a^	0.003 ± 0.003 ^b^	0.006 ± 0.0007 ^b^	0.005 ± 0.0001 ^b^	0.006 ± 0.0003 ^b^	0.007 ± 0.0005 ^b^
UFA	0.16 ± 0.025 ^a^	0.11 ± 0.001 ^b^	0.12 ± 0.01 ^b^	0.11 ± 0.004 ^b^	0.09 ± 0.020 ^c^	0.07 ± 0.004 ^c^
TFA	0.26 ± 0.043 ^a^	0.21 ± 0.003 ^b^	0.20 ± 0.002 ^b^	0.19 ± 0.006 ^b^	0.15 ± 0.035 ^c^	0.11 ± 0.00 ^d^

The results are expressed as the mean ± standard error (n = 3), and different letters in the same row indicate significant differences between samples (*p* < 0.05), and “ND” indicates that it was not detected. DS: Donkey skin, LA-YS: Low-altitude yak skin, MA-YS: Medium-altitude yak skin, HA-YS: High-altitude yak skin, PLRS: Pingliang red cattle skin, XJBS: Xinjiang brown cattle skin.

## Data Availability

The original contributions presented in the study are included in the article, further inquiries can be directed to the corresponding author.
